# The Role of Stress Echocardiography in Valvular Heart Disease

**DOI:** 10.1007/s11886-022-01765-7

**Published:** 2022-08-30

**Authors:** Rodolfo Citro, Francesca Bursi, Michele Bellino, Eugenio Picano

**Affiliations:** 1 Division of Cardiology, Cardiovascular and Thoracic Department, San Giovanni Di Dio E Ruggi d, Aragona University Hospital, Salerno, Italy; 2 Vascular Pathophysiology Unit, IRCCS Neuromed, Pozzilli, Isernia Italy; 3 Dipartimento Di Scienze Della Salute, ASST Santi Paolo E Carlo Milano, Università Degli Studi Statale Di Milano, Ospedale San Paolo, Milan, Italy; 4 Department of Medicine, Surgery, and Dentistry, University of Salerno, Salerno, Italy; 5 Institute of Clinical Physiology, National Research Council, CNR Research Campus, Via Moruzzi, 1, Building C, First floor, Room 130, 56124 Pisa, Italy

**Keywords:** Aortic stenosis, Dobutamine, Exercise, Mitral regurgitation, Stress echo, Valves

## Abstract

**Purpose of Review:**

Stress echocardiography is recommended in valvular heart disease when there is a mismatch between resting transthoracic echocardiography findings and symptoms during activities of daily living. We describe the current methodology and the evidence supporting these applications.

**Recent Findings:**

The comprehensive stress echo assessment includes valve function (gradients and regurgitation), left ventricular global systolic and diastolic function, left atrial volume, pulmonary congestion, pulmonary arterial pressure, and right ventricular function, integrated with blood pressure response with cuff sphygmomanometer, chronotropic reserve with heart rate, and symptoms.

**Summary:**

Recent guidelines recommend the evaluation of asymptomatic severe or symptomatic non-severe mitral regurgitation or stenosis with exercise stress and suspected low-flow, low-gradient severe aortic stenosis with reduced ejection fraction with low dose (up to 20 mcg, without atropine) dobutamine stress. Prospective, large-scale studies based on a comprehensive protocol (ABCDE +) capturing the multiplicity of clinical phenotypes are needed to support stress echo-driven treatment strategies.

## Introduction

Stress echocardiography is an established method for evaluating patients with coronary artery disease [1, 2]. The comprehensive approach with the ABCDE protocol has recently proved feasible and useful in patients with chronic coronary syndromes, refining phenotype identification and risk stratification in these patients with many vulnerabilities beyond coronary artery stenosis [3]. Step A with regional wall motion abnormalities detects myocardial ischemia, sometimes present even with normal coronary arteries. Step B with the 4-site simplified scan by lung ultrasound detects B-lines, a sign of pulmonary congestion, and diastolic reserve. Step C is based on volumetric echocardiography to assess left ventricular preload reserve (with end-diastolic volume changes) and left ventricular contractile reserve (with left ventricular end-systolic volume changes combined with systolic blood pressure to estimate force). Step D with Doppler-based coronary flow velocity reserve in mid-distal left anterior descending coronary artery estimates coronary microcirculatory reserve and can be obtained with satisfactory success rate also during exercise, and with excellent feasibility with vasodilator stress [3]. Step E for ECG-based chronotropic reserve assesses sympathetic cardiac autonomic reserve. Each and every step adds independent and incremental prognostic information in predicting survival in patients with chronic coronary disease [3].

The same can be true in patients with valvular heart disease (VHD), with many potential vulnerabilities beyond the stenotic or regurgitant valve. Also in these patients, the versatility of stress echo remains largely underused, to date. Nevertheless, stress echocardiography for the evaluation of VHD has a recognized potential and finds a place in the 2017 specialty recommendations of the American Society of Echocardiography and the European Association of Cardiovascular Imaging [4••] as well as in the general cardiology guidelines of the American College of Cardiology (ACC)–American Heart Association (AHA) in 2020 [5••] and European Society of Cardiology (ESC) in 2021 [6••]. As symptoms may develop slowly and indolently in chronic VHD, many patients are unaware of subtle changes in effort tolerance, even when questioned directly by their physicians. Hence, guidelines have placed renewed emphasis on the role of exercise testing to provide objective evidence of exercise capacity and symptom status [5••, 6••]. In addition, while rest Doppler echocardiography is the method of choice for assessing the severity of valvular disease, stress two-dimensional and Doppler echocardiography may be useful if there is discordance among baseline measurements or with a physical examination to assess dynamic changes in hemodynamics and may be used to guide the optimal treatment strategy [5••]. In VHD, the core ABCDE protocol is integrated and enriched (ABCDE +), as needed, by the assessment of regurgitant flows (step F), transvalvular gradients (step G), left atrial volume (step L), pulmonary pressures (step P with pulmonary artery systolic pressure, and *E*/*e*′ as a proxy of pulmonary capillary wedge pressure), and right ventricular function (step R). There are some important differences between the stress echo application in chronic coronary syndromes and in VHD (Table 1).

**Table 1:** Stress echo in chronic coronary syndromes vs valvular heart disease

	**CCS**	**VHD**
Key LV parameters	RWMA	EF, GLS
Guidelines class 1	RWMA	LF-LG AS, sec. MR, MS
Guidelines class 2	CFVR (2b)	Primary MR (2a)
Competence maintenance	> 100 cases/year	> 20 cases/year
Main symptom	Chest pain	Dyspnea
Protocol in SE2030	ABCDE	ABCDE + (FGLPR as needed)
Exercise stress	Treadmill or bike (semi-supine or upright)	Semi-supine exercise
Dobutamine max dose	Up to 40 mcg + atropine	Up to 20 mcg, no atropine
Dobutamine stress stages	3′ starting from 5 mcg/kg/min	5′ to 8′ starting from 2.5 mcg/kg/min

*CCS* chronic coronary syndromes, *CFVR* coronary flow velocity reserve, *EF* ejection fraction, *GLS* global longitudinal strain, *LV* left ventricle, *LF-LG AS* low-flow, low-gradient aortic stenosis with reduced ejection fraction, *MR* mitral regurgitation, *MS* mitral stenosis, *RWMA* regional wall motion abnormality, *SE* stress echo, *sec *secondary

In general, the assessment of VHD by stress echo is more complex, time-consuming, and technically demanding. Some parameters used for VHD, such as effective regurgitant orifice area for mitral regurgitation, are not so reproducible, with a substantial dispersion of measurements even in expert hands, at rest [7, 8]. Other parameters, such as tricuspid regurgitant jet velocity for estimation of pulmonary artery systolic pressure, are less feasible during stress and remain intrinsically flow-dependent [9, 10]. Still other parameters, such as *E*/*e*′, show only a fair correlation with pulmonary capillary wedge pressure during exercise and its technical feasibility is reduced due to wave fusion and tachycardia [11, 12].

During the last decade, the evidence base supporting the use of stress echo in VHD has not increased as expected. The consequence is that the recommendations for stress echo in VHD paradoxically but inevitably lost ground in evidence-based guidelines. For instance, an increase in mean aortic gradient with exercise by > 20 mmHg as an indication for intervention in asymptomatic severe aortic stenosis was recommended in ESC guidelines in 2012 [13] as a class of recommendation 2b (may be considered) and removed in 2017 [14] and 2021 documents [6••]. Pulmonary artery systolic pressure ≥ 60 mmHg at exercise as an indication for intervention in asymptomatic severe primary mitral regurgitation was recommended (class 2b) in ACC/AHA 2007 guidelines [15] and in the ESC guidelines in 2012 [13], but taken out in 2017 [14] and in the latest ACC/AHA 2020 [5••] and ESC 2021 [6••] documents.

## Methodology

The protocol of valve stress echocardiography execution requires a comprehensive approach, with a general framework common to all applications and some additional parameters that need to be tailored to the specific valvular disease with a dedicated focus [16]. The non-imaging parameters are essential for the safety and completeness of the study, with the imaging findings that always need to be matched with symptoms, blood pressure response, heart rate, and arrhythmias [17••]. The digital acquisition is usually made in 4 steps: rest; intermediate stage (5 min of exercise); peak stress; recovery (5′ after the end of exercise). All images and loops are stored and analyzed offline after the test, and usually, no measurements are done during image acquisition. The intermediate stage is important to assess changes in preload since the initial dilation of left ventricular end-diastolic volume is an index of preload reserve, which if impaired may contribute to the reduction in stroke volume and cardiac reserve during stress. The intermediate stage is also useful to *E*/*e*′ (before wave fusion) and to assess tricuspid regurgitant jet velocity to estimate pulmonary artery systolic pressure since sometimes the signal is lost at peak exercise and most changes occur in the early stages of exercise. The recovery phase is especially important for *E*/*e*′ (again measurable after heart rate slowing) and B-lines. B-lines require the acquisition of 1 min with the simplified 4-site scan and this lung scan can be performed in the early recovery phase without loss of information since pulmonary congestion persists after stress interruption [18, 19]. In the 4-site simplified scan, a total of 4 chest sites are scanned, symmetrically and bilaterally, on the third intercostal space in the 2 regions between the mid-axillary and anterior axillary lines and the anterior axillary and the midclavicular lines, the “wet spots” where lung water accumulates most at rest and during stress. Each site has a score from 0 to 10. The B-lines score is the sum of the score in each of the 4 chest sites (each site with a possible score from 0 to 10), generating a total score of all 4 chest zones from 0 (all 4 sites with 0 individual site scores) to 40 (all 4 sites with individual site score of 10). A number of stress B-lines are categorized as absent (score points 0–1); mild (2 to 4); moderate (5 to 9); and severe (≥ 10 points). B-lines may appear or increase during stress in the presence of mitral stenosis [20], asymptomatic severe aortic regurgitation [21], or ischemic secondary mitral regurgitation [22].

In VHD, the test protocol must be tailored to the individual patient. To ensure good quality data, we must prioritize data collected at peak stress. It is unlikely to achieve good data if there are too many measurements to be obtained at the peak. Hence, we should prioritize steps on moderate regurgitant flow and gradients as the first-tier measurement, followed by the remainder (Table 2). However, when regurgitation or stenosis is severe at rest, there is no need to measure it at peak stress and acquisition should focus on left ventricular function and pulmonary pressures [4••]. Thus, we recommend limiting peak acquisition data to no more than 6–7 items; second-tier measurement can be obtained at early recovery (B-lines and *E*/*e*′).

**Table 2:** General protocol of exercise stress echo in valvular heart disease

	**2D**	**LUS**	**CFD**	**CWD**	**PWD**	**Mmode**	**TDI**	**STE**	**ECG**	**BP**
Parameters	EDV, ESV, LAV	B-lines	PISA, EROA	TRV, MAG, MMG	*E*, VTI	TAPSE	*e*′	GLS	HR	SBP
**Steps**										
1. Rest	v	v	v	v	v	v	v	v	v	v
2. Low load	v			v					v	v
3. Peak	v		v	v	v	v		v	v	v
4. Early rec		v					v		v	v
5. Recovery	v								v	v

*BP* blood pressure, *CFD* color-flow Doppler imaging, *2D* 2-dimensional echocardiography, *ECG* electrocardiogram, *EDV* end-diastolic volume, *EF* ejection fraction, *ESV* end-systolic volume, *GLS* global longitudinal strain, *HR* heart rate, *LAV* left atrial volume, *LUS* lung ultrasound, *MAG* mean aortic gradient, *MMG* mean mitral gradient, *PISA* proximal isovelocity surface area, *PWD* pulsed-wave Doppler, *rec* recovery, *SBP* systolic blood pressure, *STE* strain echocardiography, *TAPSE* tricuspid annular plane systolic excursion, *TDI* tissue Doppler imaging, *TRV* tricuspid regurgitant jet velocity, *VTI* velocity–time integral

The best sequence of acquisition must be tailored to the individual patient and specific question. There is some simple general rule and acquisition sequence during stress. Left ventricular views with 2-dimensional echocardiography are always first. Lung ultrasound 4-site scan for B-lines (step B) is recorded at baseline and in the early recovery phase since they persist for a few minutes. The intermediate step is especially important for tricuspid regurgitant velocity jet sampling often lost at peak stress. The peak stress acquisition should focus on parameters required for valve function characterization such as gradients and regurgitation. At intermediate stages and in the early recovery phase, it is also possible to image coronary flow velocity in the mid-distal left anterior descending coronary artery to assess coronary flow velocity reserve [23]. This is feasible during semi-supine exercise and provides important prognostic information also in patients with VHD. Promising evidence is available, especially in patients with severe aortic stenosis and angiographically normal coronary arteries. In these patients, the degree of coronary microvascular disease mirrored in a reduction of coronary flow reserve during vasodilator testing is unrelated to left ventricular hypertrophy and is related to exercise capacity, is reversible after valve replacement before regression of the left ventricular hypertrophy, and predicts outcomes better than the severity of valve stenosis [24–28]. It is increasingly clear that coronary microcirculation is impaired in VHD, and it impacts myocardial remodeling, aortic flow patterns, and clinical progression in aortic stenosis [29].

## Current Approach and Guideline Recommendations

The indication of stress echo testing is always a second line after transthoracic (and sometimes transesophageal) echocardiography, necessary for the diagnosis of VHD, and to define etiology, severity, and prognosis [6••]. Expertise in echocardiography is an essential part of the heart valve team, and the upgrade to stress echo is simple. For all applications in VHD, exercise is the test of choice since it is the most physiological. Among stress exercise modalities, a semi-supine bike is recommended for obtaining Doppler data. Low-dose dobutamine (up to 20 mcg, without atropine) is the first choice in low-flow, low-gradient aortic stenosis to separate true from pseudo-severe aortic stenosis [5••, 6••]. Vasodilator stress is only used to test coronary flow velocity reserve in the left anterior descending artery, which may add to phenotyping and prognostic stratification [4••, 30••].

## Detection of Coronary Artery Disease in VHD: Stress Echo Not Indicated

The use of stress tests to detect coronary artery disease associated with a severe valvular disease is discouraged because of their low diagnostic value [6••]. This is especially true for ECG and perfusion abnormalities, but the loss in specificity also affects, to a lesser extent, regional wall motion abnormalities and stress echo [31].

## Mitral Regurgitation: Stress Echo Is Useful in Chronic Secondary and May Be Useful in Primary Forms

In chronic secondary MR (stages B to D), exercise stress echocardiography is particularly useful (class of recommendation 1 for ACC/AHA guidelines 2020) to establish the etiology of MR and to assess myocardial viability [5••]. In chronic secondary mitral regurgitation, a large increase in mitral regurgitation severity (increase in effective regurgitant orifice area ≥ 13 mm^2^) is associated with a higher risk in medically treated patients [32••]. Of note, in patients with ischemic MR (previous inferior myocardial infarction and secondary MR), the presence of a recruitable viable myocardium of the basal inferior wall is associated with a reduction of effective regurgitant orifice area. Another important finding during exercise stress echo is the increase in systolic arterial pulmonary pressure [32••] and the appearance of exercise-induced B-lines [33]. Exercise pulmonary hypertension is a sign of systemic hemodynamic congestion, and B-lines are a sign of pulmonary congestion. Both signs are independently associated with more cardiac events.

**Fig. 1:**
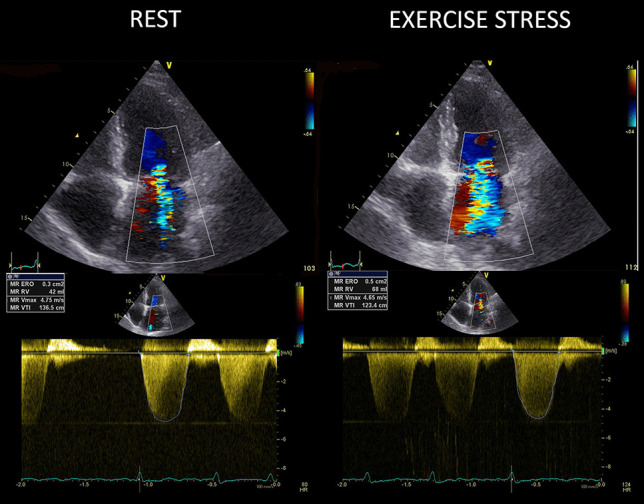
The worsening MR pattern during stress. Apical 4-chamber view showing color-flow Doppler (upper panels) and continuous wave Doppler (lower panels) at rest (left panels) and at peak exercise (right panels) in a patient with a large exercise-induced increase in mitral regurgitation. ERO, effective regurgitant orifice; RVol, regurgitant volume. ERO increases from 0.3 cm^2^ at rest (moderate) to 0.5 cm^2^ at peak stress (severe). Courtesy of Dr. Angela Zagatina, Saint Petersburg, Russia, part of the Stress Echo 2030 study group

In patients with primary mitral regurgitation (stages B and C) and symptoms that might be attributable to mitral regurgitation, hemodynamic exercise testing using Doppler echocardiography or cardiac catheterization or cardiopulmonary exercise testing is reasonable (class of recommendation 2a) [5••]. In patients with symptoms but mild or moderate mitral regurgitation at rest, an assessment of MR should be performed to evaluate exercise tolerance, changes in mitral regurgitation (Fig. 1), and contractile reserve.

In primary MR, normal mitral annulus dynamic function is strongly altered. This condition represents a predisposing factor to significant changes in regurgitant volume during the cardiac cycle. There is a good correlation between exercise-induced changes in the mitral valve annulus area and the changes in mitral regurgitant volume. Marked changes in MR severity of at least 1 grade [4••] are associated with exercise-induced changes in systolic pulmonary arterial pressure and reduced symptom-free survival [34].

Moreover, during stress echocardiography, contractile reserve and changes in global longitudinal strain should both help in stratifying the prognosis of patients with MR. The assessment of exercise pulmonary artery systolic pressure (> 60 mmHg) and left ventricular function (abnormal response defined by Δ left ventricular ejection fraction < 5% or Δ global longitudinal strain < 2%) may provide additional information on symptomatic and asymptomatic MR, but are not, actually, endorsed by guidelines [34]. Furthermore, the onset of right ventricular dysfunction, evaluated as tricuspid annular plane systolic excursion < 19 mm, might help to stratify prognosis independently of the onset of pulmonary hypertension, in asymptomatic primary MR [35]. In asymptomatic chronic primary mitral regurgitation, exercise-induced pulmonary hypertension is no longer recommended as a criterion for intervention [5••, 6••].

## Mitral Stenosis: Stress Echo May Be Useful

In rheumatic mitral stenosis and a discrepancy between resting echocardiographic findings and clinical symptoms, exercise testing with Doppler is recommended to evaluate symptomatic response, exercise capacity, and the response of the mean mitral gradient and pulmonary artery pressure [5••]. The abnormal response is identified as an increase in the mean transmitral gradient ≥ 15 mmHg or estimated pulmonary artery systolic pressure ≥ 60 mmHg [4••]. In symptomatic patients with rheumatic mitral stenosis and a mitral valve area > 1.5 cm^2^, if there is evidence of hemodynamically significant rheumatic mitral stenosis on the basis of a pulmonary artery wedge pressure > 25 mmHg or a mean mitral valve gradient > 15 mmHg during exercise, percutaneous mitral commissurotomy may be considered if it can be performed at a Comprehensive Valve Center (class of recommendation 2b) [5••].

In patients with ambiguous symptoms that are suspected to be attributable to mixed mitral valve disease, further assessment of filling pressure by using biomarkers or invasive hemodynamic measurements at rest or with exercise is reasonable (class of recommendation 2a). The invasive hemodynamic measurement is recommended. Noninvasive assessment with stress echo may supply ancillary information showing a pulmonary artery systolic pressure ≥ 60 mmHg or B-lines during stress as a sign of pulmonary congestion pointing to a cardiac origin of symptoms [4••].

Stress echo is especially useful in patients with valve prostheses and uncovers hemodynamic changes in prosthetic valves in patients with a discrepancy between symptoms and valvular disease severity (stenosis or regurgitation). A disproportionate increase in mean transvalvular gradient either during exercise (i.e., > 20 mmHg for aortic prostheses or > 12 mmHg for mitral prostheses) suggests severe prosthesis stenosis or significant patient-prosthesis mismatch [4••].

## Aortic Stenosis: Stress Echo Is Recommended or Is Contraindicated, Depending on the Specific Phenotype

Severe aortic stenosis is defined as an aortic valve area ≤ 1.0 cm^2^ compared to the normal value of 3.0–4.0 cm^2^. The reduction of the aortic valve area must be substantial before the pathologic disease becomes hemodynamically significant and, at a later stage, clinically significant. In parallel, a minimal valve gradient is present until the orifice area becomes less than half of normal. The pressure gradient across the valve is directly related to the valve orifice area and the transvalvular flow. The pragmatic consequence of this simple hydraulic principle is that complete assessment of aortic stenosis (and any valve stenosis) requires, at rest and during stress, the measurement of the transvalvular flow, transvalvular pressure gradient, and calculation of the aortic valve area. If one of the three is missing, the study is incomplete. Apart from the relatively rare occurrence of a high flow status condition, a high transvalvular gradient at rest is diagnostic for severe aortic stenosis [5••]; conversely, a low gradient at rest in the presence of a reduced aortic valve area cannot rule out the presence of severe aortic stenosis. A normal aortic valve area at rest excludes severe aortic stenosis, but a reduced aortic valve area is not sufficient to confirm severe aortic stenosis in the presence of a reduced left ventricular ejection fraction and/or reduced transvalvular flow. According to transvalvular flow, ejection fraction, and transvalvular pressure gradients, patients with reduced aortic valve area are grouped into 4 broad categories identified by guidelines [6••]: high gradient aortic stenosis; low-flow, low-gradient aortic stenosis with reduced ejection fraction; low-flow, low-gradient aortic stenosis with preserved ejection fraction; normal-flow, low-gradient aortic stenosis with preserved ejection fraction.

Exercise stress echocardiography does not have a specific indication in asymptomatic patients with severe aortic stenosis (stage C1, aortic velocity ≥ 4.0 m/s or mean pressure gradient ≥ 40 mmHg), although exercise testing is quoted in the guidelines as it provides diagnostic and prognostic information. A fall in systolic blood pressure (≥ 20 mmHg in ACC/AHA and > 20 mmHg in ESC document, respectively) and decreased exercise tolerance are indications of intervention (class 2a for both 2020 ACC/AHA and 2021 ESC guidelines). According to the 2021 ESC guidelines, intervention should be considered in asymptomatic patients with severe aortic stenosis (class of recommendation 2a) in the presence of resting left ventricular systolic dysfunction (ejection fraction < 55%) without another cause and is recommended if ejection fraction < 50% (class of recommendation 1) [6••]. Stress testing with exercise is useful to identify patients who are truly asymptomatic since the underestimation of symptoms is common in VHD with patients limiting their level of activity over the years to adapt to the slow progression of valve disease. Stress testing is not indicated and can be harmful (contraindicated, class 3) in symptomatic patients with severe aortic stenosis (stage D1, aortic velocity ≥ 4.0 m/s or mean pressure gradient ≥ 40 mmHg), because of the risk of severe hemodynamic compromise, with a rate of 1 in 500 of major complications including severe hypotension, cardiac asystole, ventricular tachycardia, and death [5••]. According to the latest ACC/AHA 2020 guidelines, “recording valve hemodynamics during exercise in aortic stenosis is of limited value and does not show additive value for predicting clinical outcome when baseline measures of hemodynamic severity and functional status are considered” [5••].

## Dobutamine Stress Echo Is Recommended in Low-Flow, Low-Gradient Aortic Stenosis with Reduced Ejection Fraction

In patients with suspected low-flow, low-gradient severe aortic stenosis with reduced ejection fraction (mean gradient < 40 mmHg, valve area ≤ 1 cm^2^, left ventricular ejection fraction < 50%, stroke volume index ≤ 35 mL/m^2^), low-dose dobutamine stress echocardiography is recommended by ESC guidelines (class 1) to distinguish between true severe and pseudo-severe aortic stenosis (increase in valve area to > 1.0 cm^2^ with the increased flow) and identify patients with no flow (or contractile) reserve [6••]. Low-dose dobutamine stress testing with either echocardiographic or invasive hemodynamic measurements is considered reasonable (class 2a) to further define severity and assess contractile reserve for ACC/AHA 2020 guidelines [5••]. A stress echocardiogram with low-dose dobutamine is used in these patients to assess left ventricular flow (contractile) reserve and aortic valve stenosis severity by measurement of aortic valve area by continuity equation at baseline and during the infusion of the drug [6••]. Three response patterns are possible.

**Fig. 2:**
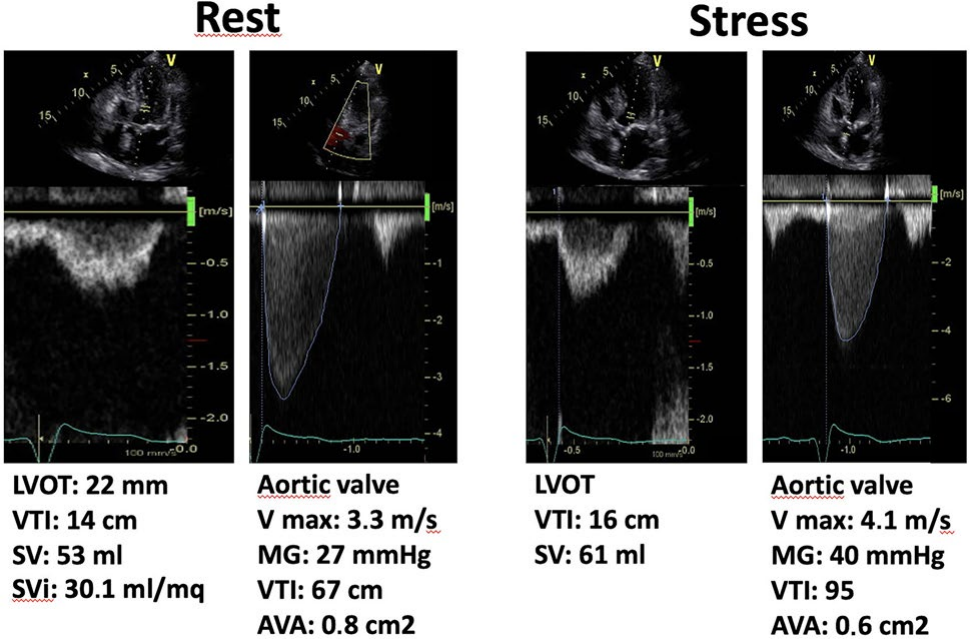
Low-flow, low-gradient aortic stenosis at rest (on the left), in a patient with low ejection fraction which turns into truly severe aortic stenosis during dobutamine stress echocardiography at the low dose of 10 μg/kg/min (on the right). AVA, aortic valve area; LVOT, left ventricle outflow tract; MG, mean gradient; SV, stroke volume; Svi, stroke volume indexed; VTI, velocity time integral. Courtesy of Dr. Rodolfo Citro, Salerno, Italy, part of the Stress Echo 2030 study group

During dobutamine, the typical pattern of pseudo-severe aortic stenosis is the increase in flow reserve (≥ 20% stroke volume increase) with a significant increase in aortic valve area (to > 1cm^2^) and a minor increase in transvalvular gradient. Medical therapy optimization and resting echocardiographic follow-up are recommended. The typical pattern of true severe aortic stenosis is the increase in stroke volume (increase ≥ 20%) with an unchanged aortic valve area (≤ 1cm^2^) and a marked increase in transvalvular gradient (mean pressure gradient ≥ 40 mmHg or peak aortic jet velocity ≥ 4 m/s) at any flow (Fig. 2). In these patients, intervention is recommended, with a class of recommendation 1 for ESC 2021 guidelines [6••].

The third pattern of indeterminate aortic stenosis severity occurs in patients with a lack of flow reserve (increase in indexed stroke volume ≤ 20%) with unchanged aortic valve area, and unchanged transvalvular gradient. The absence of contractile reserve occurs in up to 30% of patients and is a predictor of high perioperative mortality after surgical aortic valve replacement. However, because this pattern does not predict late post-intervention survival, it should not contraindicate surgical or percutaneous interventions, which improve long-term prognosis and favor left ventricular function improvement. In this group, intervention should be considered with a class of recommendation 2a for ESC 2021 guidelines, especially when cardiac computed tomography calcium scoring confirms severe aortic stenosis [6••].

Applications of potential value have been suggested for other conditions. In asymptomatic chronic severe aortic regurgitation, the timing of intervention could be closer in the presence of a lack of contractile reserve of the left ventricle (increase in ejection fraction < 5%).

## Evidence Gaps

Despite the enormous potential information to be gained, stress echo lacks robust supportive evidence in the particular field of VHD. Most recommendations are based on the class of evidence C (“consensus opinion of experts and/or small, retrospective studies”), with a minority with the level of evidence B (“large non-randomized studies, single randomized clinical trial”), and no recommendation rated as A (“multiple randomized studies or meta-analysis”). Stress echo parameters such as pulmonary artery systolic pressure > 60 mmHg on exercise are used with binary cutoff values as a possible indication for intervention, but the evidence supporting this crucial decision is weak, and therefore some areas, such as asymptomatic severe mitral regurgitation, are prioritized for research recommendations by a November 2021 document of the National Institute for Health and Care Excellence in the UK [36•].

Some of these applications are not so easy to execute and may not be safe if performed outside of a dedicated and experienced stress echo laboratory [37•], and yet the recommended caseload for a level III echo competency includes 200 stress echo studies per year of which 25 need to be non-coronary indications [38], meaning that a laboratory can perform only 1 or 2 stress echo studies per year in low-flow low-gradient aortic stenosis with reduced ejection fraction and remains competent.

## Conclusion

Stress echocardiography with exercise or dobutamine has important applications in the assessment of VHD. Its potential is however underused. The comprehensive approach with the ABCDE protocol has already proved feasible and useful in patients with chronic coronary syndromes, refining phenotype identification and risk stratification in these patients with many vulnerabilities beyond coronary artery stenosis [3]. The same can be true in patients with VHD, with many potential vulnerabilities beyond the stenotic or regurgitant valve. Step A with regional wall motion abnormalities detects myocardial ischemia, sometimes present even with normal coronary arteries. Step B with the simplified 4-site scan by lung ultrasound detects B-lines, a sign of pulmonary congestion, and diastolic reserve. Step C is based on volumetric echocardiography to assess left ventricular preload reserve (with end-diastolic volume changes) and left ventricular contractile reserve (with left ventricular end-systolic volume changes combined with systolic blood pressure to estimate force). Step D with Doppler-based coronary flow velocity reserve in mid-distal left anterior descending coronary artery estimates coronary microcirculatory reserve and can be obtained with satisfactory success rate also during exercise, and with excellent feasibility with vasodilator stress [3]. Step E for ECG-based chronotropic reserve assesses sympathetic cardiac autonomic reserve. Each and every step adds independent and incremental prognostic information in predicting survival in patients with chronic coronary disease [3]. The comprehensive stress echo approach with the ABCDE protocol has been adopted and disseminated in the large-scale, multicenter, international, prospective stress echo 2030 study which networks the experience of > 30 centers from > 20 countries [39]. The study will collect, by the year 2025, 10,000 patients spread over 12 different protocols, also including patients with VHD. As of June 30th, 2022, over 3500 patients have been stored in the data bank owned by the Italian Society of Echocardiography and Cardiovascular Imaging. In VHD, the core ABCDE protocol is integrated and enriched (ABCDE +), as needed, by the assessment of regurgitant flows (step F), transvalvular gradients (step G), left atrial volume (step L), pulmonary pressures (step P with pulmonary artery systolic pressure, and *E*/*e*′ as a proxy of pulmonary capillary wedge pressure), and right ventricular function (step R). With this comprehensive methodological platform, the subprojects SEMIR (subproject 10, stress echo in mitral ischemic regurgitation) and SEVA (subproject 11, stress echo in valvular heart disease) of stress echo 2030 study plan to recruit 500 patients each by the year 2025, and follow-up will continue until 2030. The rationale behind the study is simple. There is more than a diseased valve in VHD, and a comprehensive stress echo approach may be suitable for the task of capturing the multiplicity of clinical phenotypes, prognostic vulnerabilities, and potential therapeutic targets of these challenging patients [40].
